# Efficacy and Safety of Intra-Articular Platelet-Rich Plasma in Osteoarthritis Knee: A Systematic Review and Meta-Analysis

**DOI:** 10.1155/2021/2191926

**Published:** 2021-04-30

**Authors:** Mao Hong, Chongjie Cheng, Xiaowei Sun, Yan Yan, Qidong Zhang, Weiguo Wang, Wanshou Guo

**Affiliations:** ^1^Beijing University of Chinese Medicine, Beijing 100029, China; ^2^Department of Orthopaedic Surgery, Beijing Key Lab Immune-Mediated Inflammatory Diseases, China-Japan Friendship Hospital, Beijing 100029, China; ^3^Graduate School of Peking Union Medical College, Chinese Academy of Medical Sciences, Beijing 100029, China; ^4^Peking University China-Japan Friendship School of Clinical Medicine, 2 Yinghuadong Road, Chaoyang District, Beijing 100029, China

## Abstract

**Background:**

Knee osteoarthritis (KOA) is a common disease in aged adults. Intra-articular (IA) injection of platelet-rich plasma (PRP) therapy is an effective minimally invasive treatment for KOA. We aimed to compare the efficacy and safety of platelet-rich plasma (PRP) with placebo or other conservative treatments.

**Methods:**

We conducted a meta-analysis to identify relevant articles from online register databases such as PubMed, Medline, Embase, and the Cochrane Library. The primary outcomes were the visual analogue scale (VAS) score, Western Ontario and McMaster Universities Arthritis Index (WOMAC) score, and International Knee Documentation Committee (IKDC) subjective score. The secondary outcome was the adverse event rate.

**Results:**

A total of 895 articles were identified, of which 23 randomized controlled trials that met the inclusion criteria were determined as eligible. Compared with placebo, PRP had a lower VAS score and higher IKDC subjective score at the 6^th^ month after treatment and significantly less WOMAC score during the follow-up period. Compared with oral NSAIDs, PRP gained a lower WOMAC score at the 6^th^ month after treatment. The VAS score decreased after treatment when reaching PRP and CS. As compared to the HA, the VAS score, WOMAC score, and IKDC subjective score all revealed better PRP results. There were no significant differences in adverse event rates comparing PRP versus placebo or HA. Different PRP applications did not show significant differences in VAS score in the 1^st^ month and WOMAC score in the 3^rd^ month after treatment.

**Conclusion:**

To compare with the conservative treatments mentioned above, PRP is more effective in relieving symptoms. There were no significant differences between triple PRP application and single PRP application in short-term curative effect.

## 1. Introduction

Knee osteoarthritis is prevalent globally among the aged adults with an ageing and increasingly obese population [1]. It is also the second leading cause of disability and a heavy economic and social burden [2]. Pain, swelling, stiffness, and limitation of motion are the most frequent symptoms in patients who suffered from KOA. KOA management includes conservative treatment such as patient education, weight loss, exercise, pain medication, intra-articular hyaluronan, glucosamine or chondroitin, and joint replacement surgery for end-stage patients. Arthroplasty surgery is a clinically relevant and cost-effective treatment for end-stage KOA. However, it can only be considered cost-effective if the procedure is restricted to patients with more severely affected functional status [1].

Oral NSAIDs are effective in terms of clinically relevant improvement of both pain and function, intra-articular corticosteroids are recommended for knee osteoarthritis for patients who have not responded to oral or topical analgesics [1], and the clinical efficacy of intra-articular HA injection for the treatment of OA knees also has beneficial effects on pain, function, and global patient assessment [3].

Platelet-rich plasma (PRP) concentrates a high number of platelets in a small volume of plasma, and it is prepared by centrifugation of autologous blood [4]. Generally, PRP is prepared with two centrifugations, including separating erythrocytes in the first spin and concentrating platelets from the second spin [5, 6]. Intra-articular injection of PRP has occurred as a disease modification therapy in recent years since it had been proved as a simple, low-cost, and minimally invasive therapy that provides a natural concentrate of autologous blood growth factors that can be used to enhance tissue regeneration [5]. In addition to the release of growth factors, PRP also promotes concentrated anti-inflammatory signals, including interleukin-1ra, which focuses on emerging treatment methods for osteoarthritis [7]. The main advantages of platelet concentrates are their low cost, since their preparation through a simple centrifugation process, and the fact that they are obtained from the patient's blood [8]. But its clinical safety or efficacy on pain decrease and function promotion is still controversial, especially compared with other traditional conservative therapy, including oral NSAIDs, intra-articular CS or HA, and even placebo. Furthermore, there is no consensus on the number and frequency of injections. Hence, the effect of different PRP applications on pain and physical function in knee osteoarthritis is also assessed in this study.

Besides, some scholars use single centrifugation to prepare PRP [9–11], and a few researchers apply leucocyte filters to reduce the leucocyte count in their studies [9]. Therefore, the clinical outcomes may be affected due to the preparation and formulation of PRP. We further analyzed the relevant data in the included studies, attempting to find out how the results varied based on PRP's formulation.

## 2. Materials and Methods

### 2.1. Search Strategy

The systematic review was structured to adhere to the Preferred Reporting Items for Systematic Reviews and Meta-Analyses (PRISMA) Statement reporting guidelines for the meta-analysis of intervention trials (Supplementary Data Set 1). Ethical approval was not needed because all the data presented in this study were extracted from published articles and did not cover any personal data. Clinical trials that compared intra-articular PRP injection with oral NSAIDs, intra-articular HA injection, intra-articular CS injection, or application of placebo for pain and function management in patients who suffered from KOA were identified. Online register databases, including Medline, PubMed, Embase, and the Cochrane Library, were searched until December 2019. The following search terms were used: “Osteoarthritis, Knee” OR “Knee Osteoarthritis” OR “Knee Osteoarthritis” OR “Osteoarthritis Of Knee” OR “Knee, Osteoarthritis Of” OR “Knees, Osteoarthritis Of” OR “Osteoarthritis Of Knees” AND “Platelet-rich plasma” OR “PRP.” Publication language was limited to English. References within included articles were manually searched for any additional trials.

### 2.2. Eligibility Criteria and Study Selection

#### 2.2.1. The Selection Criteria Used for our Meta-Analysis Are Listed below

The inclusion criteria according to the PICOS criteria were studies including the following:

1. *Population*. Patients suffered from KOA.
2. *Intervention*. Intra-articular injection of PRP.
3. *Comparator*. Oral NSAIDs, intra-articular injection of PRP, intra-articular injection of HA, intra-articular injection of CS, intra-articular injection of a placebo, or different frequency of PRP application.
4. *Outcomes*. The primary outcomes included 10 mm visual analogue scale (VAS) (scale 0–10, where 0 = no pain and 10 = worst imaginable pain) score, 100 mm VAS (scale 0–100, where 0 = no pain and 100 = worst imaginable pain) score, Western Ontario and McMaster Universities Arthritis Index (WOMAC) score, and International Knee Documentation Committee (IKDC) subjective score at preinjection, 1^st^ month, 2^nd^ month, 3^rd^ month, 6^th^ month, or 12^th^ month after treatment. The secondary outcome was the adverse event rate.
5. *Study Design*. Randomized controlled trials (RCTs).

#### 2.2.2. The Exclusion Criteria Were Studies That Were

1. Reviews
2. Case reports
3. Non-RCT trials or nonhuman trials

### 2.3. Data Extraction

Two authors independently reviewed the full text of the selected studies. Raw information, including author, publication year, study design, population, gender, age, intervention, primary outcomes, and secondary outcome, were extracted. The primary products included 10 mm visual analogue scale (VAS) (scale 0–10, where 0 = no pain and 10 = worst imaginable pain) score, 100 mm VAS (scale 0–100, where 0 = no pain and 100 = worst imaginable pain) score, Western Ontario and McMaster Universities Arthritis Index (WOMAC) score, and International Knee Documentation Committee (IKDC) subjective score at baseline, 1^st^ month, 2^nd^ month, 3^rd^ month, 6^th^ month, or 12^th^ month after treatment. The secondary outcome was the adverse event rate. For studies with incomplete data, we contacted the authors to ensure the integrity of the data.

### 2.4. Quality Evaluation

**Figure 1:**
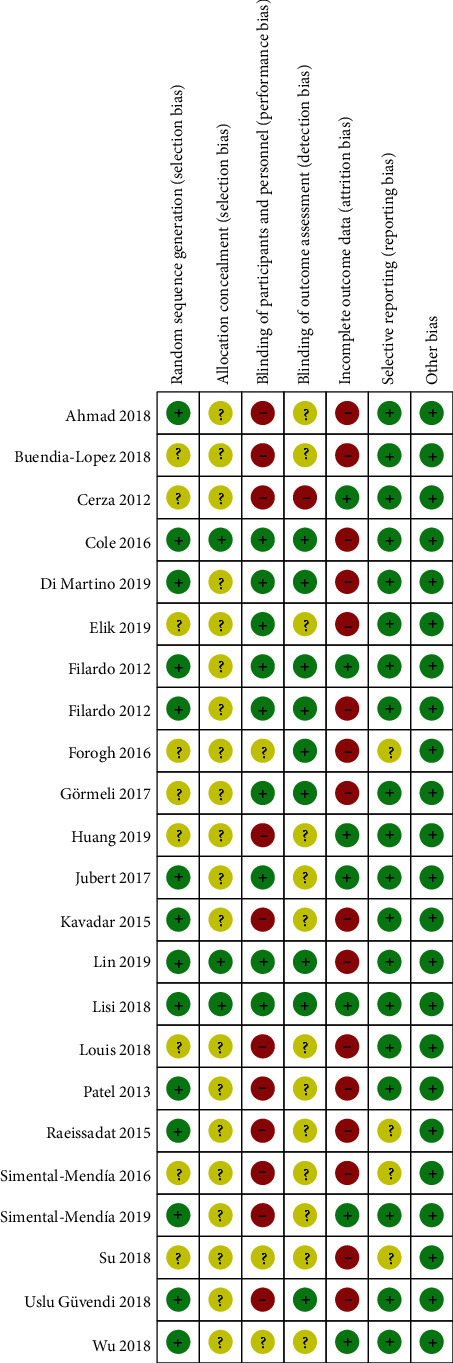
Results of the methodological quality evaluations. Green indicates that the criterion is satisfied. Yellow indicates that it is unclear whether the criterion is satisfied or not. Red indicates that the study did not meet the criterion.

We followed the methods of Wei Zuo et al. to conduct a study quality assessment because of their scientific methodology [12]. The methodological quality of randomized controlled trials (RCTs) was assessed by a modified version of the Jadad Scale (0 [“very poor”] to 7 [“rigorous”]). The modified version of the Jadad Scale includes four domains: randomization, concealment of allocation, double-blinding, withdrawals, and dropouts (Figure 1). The higher the score, the better was the quality of the article. We also conducted a sensitivity analysis to evaluate whether any single study had the weight to skew on the overall estimate and data. Furthermore, we did not conduct publication bias because of the limited number of included studies. Two authors independently performed the assessment, and disagreements were resolved by discussion.

### 2.5. Statistical Analysis and Data Synthesis

Calculations of this meta-analysis were performed using the Review Manager Software (Revman v5.3, Copenhagen: The Nordic Cochrane Centre, the Cochrane Collaboration). The continuous outcomes, including VAS score, WOMAC score, and IKDC subjective score, were assessed using mean difference (MD) or stand mean difference (SMD) with 95% confidence intervals (CI). The dichotomous outcome (adverse event rate) was assessed using relative risks (RR) with 95% CI. *P* value <0.05 was considered to be statistically significant.

### 2.6. Investigation of Heterogeneity

Heterogeneity among the studies was assessed using the chi-square test based on the *P* and *I*^2^ values. *I*^2^ > 50% indicated substantial heterogeneity. Therefore, a random effect model was used to assess the outcome. If substantial heterogeneity remained, subgroup analysis was used to interpret the potential source of heterogeneity. Since the importance of inconsistency depends on several factors, interpreting the threshold of *I*^2^ may be misleading. *I*^2^ < 50% and *P* > 0.1 indicate that the heterogeneity may not be important, and a fixed-effect model was used to evaluate the outcome.

## 3. Results

### 3.1. Data Selection and Study Characteristics

**Figure 2:**
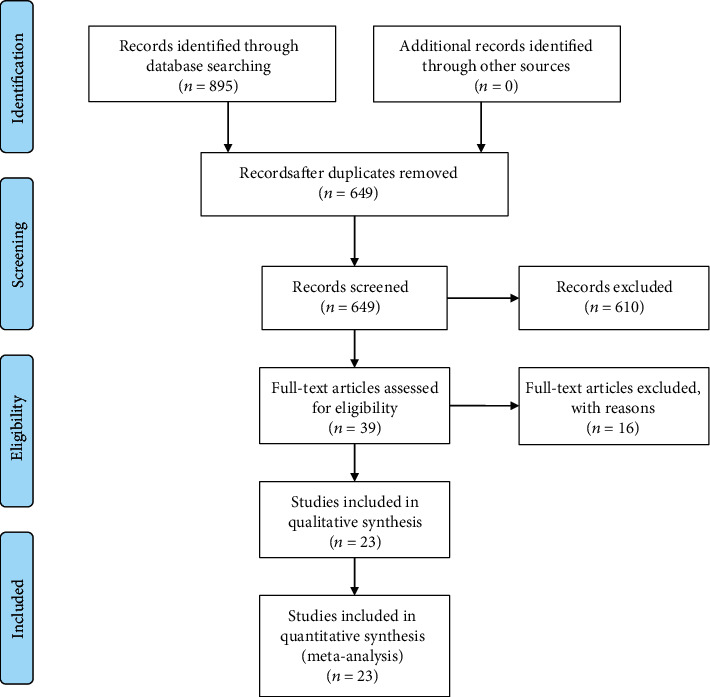
PRISMA flowchart. A total of 895 studies were evaluated. Titles and abstracts were assessed, and 39 full-text articles were eligible for evaluation. Sixteen articles were excluded, and 23 articles remained for the final analysis.

A total of 895 articles were initially identified from online register databases by keyword search, and 856 articles were excluded after the primary review of the titles and abstracts. Full texts of the remaining 39 articles were evaluated, and 16 articles that did not meet the inclusion criteria were excluded. Finally, 23 articles with a total of 2222 patients (including 2355knees) met the selection criteria and were determined as eligible (Figure 2) [3, 4, 6, 8–11, 13–28]. They were all randomized controlled trials (RCTs). All the included articles were in English and were published between 2012 and 2019. The characteristics of the 23 included articles are presented in Table 1.

### 3.2. Meta-Analysis

#### 3.2.1. IA PRP versus IA Placebo (Saline Solution)

**Figure 3:**
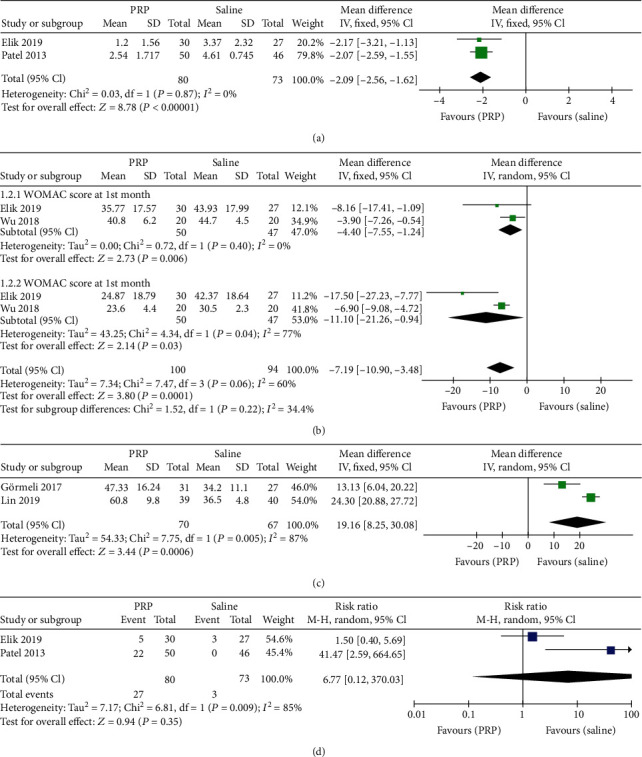
Forest plot analysis of (a) VAS score, (b) WOMAC score, (c) IKDC subjective score, and (d) adverse events between PRP and saline.

Five studies [3, 9, 17, 20, 26] had applied intra-articular saline solution injection as a placebo to compare the efficacy and safety of intra-articular PRP injection. Two studies [9, 26] on 153 patients reported the VAS score at the 6^th^ month after treatment. The PRP group was associated with a lower VAS score at the 6^th^ month after treatment than the saline group (MD = −2.09, 95% CI: -2.56 to -1.62; *P* < 0.05; Figure 3). Two studies [3, 20] on 97 patients reported the WOMAC score at the 1^st^ month after treatment. The PRP group was associated with a lower WOMAC score at the 1^st^ month after treatment than the saline group (MD = −4.40, 95% CI: -7.55 to -1.24; *P* < 0.05; Figure 3). Two studies [3, 20] on 97 patients reported the WOMAC score at the 6^th^ month after treatment. The PRP group was associated with a lower WOMAC score at the 6^th^ month after treatment than the saline group (MD = −11.10, 95% CI: -21.26 to -0.94; *P* < 0.05; Figure 3). Two studies [3, 17] on 137 patients reported the IKDC subjective score at the 6^th^ month after treatment. The PRP group was associated with a higher IKDC subjective score at the 6^th^ month after treatment than the saline group (MD = 19.16, 95% CI: -8.25 to 30.08; *P* < 0.05; Figure 3). Two studies [9, 26] on 153 patients reported adverse events after treatment. There were no significant differences between the two groups (MD = 6.77, 95% CI: 0.12 to 370.03; *P* = 0.35; Figure 3).

#### 3.2.2. IA PRP versus Oral NSAIDs

Two studies [8, 23] had applied oral NSAIDs as a control group to compare the efficacy of intra-articular PRP injection. The two studies on 131 patients reported the WOMAC score at the 6^th^ month after treatment. The PRP group was associated with a lower WOMAC score at the 6^th^ month after treatment than the NSAID group (MD = −9.05, 95% CI: -9.58 to -8.51; *P* < 0.05; Figure S1).

#### 3.2.3. IA PRP versus IA CS

Four studies [10, 16, 22, 28] had applied intra-articular corticosteroid injection as the control group to compare intra-articular PRP injection efficacy. Two studies [16, 22] on 79 patients reported the VAS score at the 2^nd^ month after treatment. There were no significant differences between the two groups (SMD = −2.08, 95% CI: -4.45 to -0.28; *P* = 0.08; Figure S2). Three studies [10, 16, 22] on 144 patients reported the VAS score at the 6^th^ month after treatment. The PRP group was associated with a lower VAS score at the 6^th^ month after treatment than the CS group (SMD = −1.51, 95% CI: -2.87 to -0.15; *P* < 0.05; Figure S2). Two studies [22, 28] on 111 patients reported the WOMAC score at the 6^th^ month after treatment. There were no significant differences between the two groups (MD = −9.65, 95% CI: -21.35 to 2.04; *P* = 0.11; Figure S3).

#### 3.2.4. IA PRP versus IA HA

**Figure 4:**
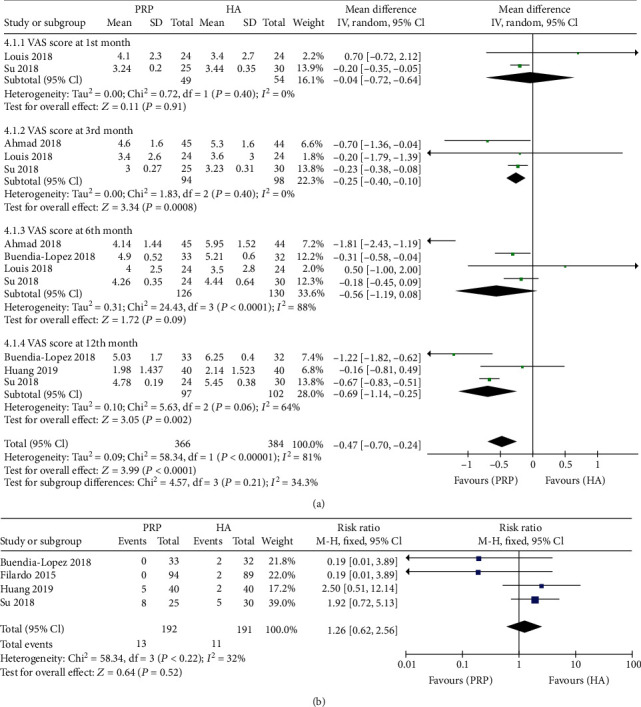
Forest plot analysis of (a) VAS score and (b) adverse events between PRP and HA.

Fourteen studies [3, 6, 11, 13–15, 17–19, 21, 23–26, 28] had applied intra-articular hyaluronic acid injection as the control group to compare the efficacy and safety of intra-articular PRP injection. Two studies [21, 24] on 103 patients reported the VAS score at the 1^st^ month after treatment. There were no significant differences between the two groups (MD = −0.04, 95% CI: -0.72 to 0.64; *P* = 0.91; Figure 4). Three studies [11, 21, 24] on 192 patients reported the VAS score at the 3^rd^ month after treatment. The PRP group was associated with a lower VAS score at the 3^rd^ month after treatment than the HA group (MD = −0.25, 95% CI: -0.40 to -0.10; *P* < 0.05; Figure 4). Four studies [11, 21, 23, 24] on 256 patients reported the VAS score at the 6^th^ month after treatment.

There were no significant differences between the two groups (MD = −0.56, 95% CI: -1.19 to 0.08; *P* = 0.09; Figure 4). Three studies [21, 23, 28] on 199 patients reported the VAS score at the 12^th^ month after treatment. The PRP group was associated with a lower VAS score at the 12^th^ month after treatment than the HA group (MD = −0.69, 95% CI: -1.14 to -0.25; *P* < 0.05; Figure 4).

Three studies [13, 21, 24] on 223 patients reported the WOMAC score at the 1^st^ month after treatment. There were no significant differences between the two groups (MD = −8.55, 95% CI: -26.69 to 9.95; *P* = 0.36; Figure S4). Four studies [13, 21, 24, 28] on 303 patients reported the WOMAC score at the 3^rd^ month after treatment. There were no significant differences between the two groups (MD = −4.96, 95% CI: -10.18 to 0.25; *P* = 0.06; Figure S4). Five studies [13, 21, 23, 24, 28] on 368 patients reported the WOMAC score at the 6^th^ month after treatment. The PRP group was associated with a lower WOMAC score at the 6^th^ month after treatment than the HA group (MD = −7.54, 95% CI: -10.54 to -4.54; *P* < 0.05; Figure S4). Four studies [15, 21, 23, 28] on 360 patients reported the WOMAC score at the 12^th^ month after treatment. The PRP group was associated with a lower WOMAC score at the 12^th^ month after treatment than the HA group (MD = −8.48, 95% CI: -12.13 to -4.83; *P* < 0.05; Figure S4).

Four studies [3, 6, 14, 25] on 519 patients reported the IKDC subjective score at the 2^nd^ month after treatment. There were no significant differences between the two groups (MD = 0.46, 95% CI: -2.31 to 3.23; *P* = 0.75; Figure S5). Six studies [3, 6, 11, 14, 17, 18] on 618 patients reported the IKDC subjective score at the 6^th^ month after treatment. The PRP group was associated with a higher IKDC subjective score at the 6^th^ month after treatment than the HA group (MD = 7.96, 95% CI: 4.46 to 11.46; *P* < 0.05; Figure S5). Four studies [3, 6, 14, 18] on 451 patients reported the IKDC subjective score at the 12^th^ month after treatment. The PRP group was associated with a higher IKDC subjective score at the 12^th^ month after treatment than the HA group (MD = 6.95, 95% CI: 1.39 to 12.50; *P* < 0.05; Figure S5).

Four studies [6, 21, 23, 28] on 383 patients reported adverse events after treatment. There were no significant differences between the two groups (MD = 1.26, 95% CI: 0.62 to 2.56; *P* = 0.52; Figure 4).

#### 3.2.5. Triple IA PRP versus Single IA PRP

**Figure 5:**
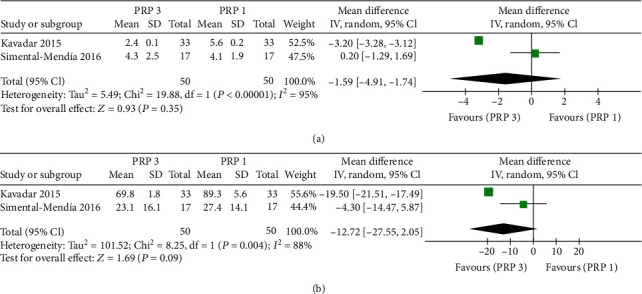
Forest plot analysis of (a) VAS score at 1st month and (b) WOMAC score at 3rd month between triple PRP and single PRP.

Two studies had compared triple intra-articular PRP injection versus single intra-articular PRP injection. There were no significant differences between the two groups on VAS score at 1^st^ month after treatment (MD = −1.59, 95% CI: -4.91 to 1.74; *P* = 0.35; Figure 5) and WOMAC score at 3^rd^ month after treatment (MD = −12.75, 95% CI: -27.55 to 1.74; *P* = 2.05; Figure 5).

#### 3.2.6. Sensitivity Analysis

Among the outcomes with high heterogeneity, the sensitivity analysis showed that excluding any one single study did not change the statistical results. Therefore, we believe that our findings in this review are reliable.

#### 3.2.7. Subgroup Analysis

**Figure 6:**
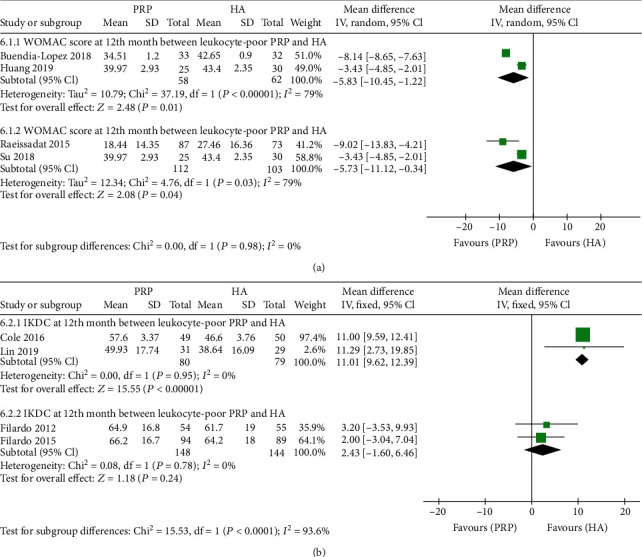
Subgroup forest plot analysis of (a) WOMAC score and (b) IKDC subjective score between LP-PRP or LR-PRP and HA.

To investigate the influence of the cellular composition of PRP, we conducted a subgroup analysis to identify whether leukocyte-poor PRP (LP-PRP) was distinguishing from leukocyte-rich PRP (LR-PRP) in comparing the efficacy with HA for KOA treatment (Figure 6). Two studies [23, 28] reported the WOMAC score at 12^th^ month between the LP-PRP and HA, and pooled results revealed that the LP-PRP group was associated with a lower WOMAC score at 12^th^ month after treatment than the HA group (MD = −5.83, 95% CI: -10.45 to -1.22; *P* = 0.01; Figure 6(a)). Two studies [15, 21] reported the WOMAC score at the 12^th^ month between the LR-PRP and HA. The LR-PRP group was also associated with a lower WOMAC score at the 12^th^ month after treatment than the HA group (MD = −5.73, 95% CI: -11.12 to -0.34; *P* = 0.04; Figure 6(a)). Two studies [3, 18] reported the IKDC subjective score at 12^th^ month between the LP-PRP and HA, and pooled results revealed that the LP-PRP group was associated with a higher IKDC subjective score at the 12^th^ month after treatment than the HA group (MD = 11.01, 95% CI: 9.62 to 12.39; *P* < 0.00001; Figure 6(b)). Two studies [6, 14] reported the IKDC subjective score at 12^th^ month between the LR-PRP and HA, but pooled results revealed that there were no significant differences between the two groups (MD = 2.43, 95% CI: -1.60 to 6.46; *P* = 0.24; Figure 6(b)).

## 4. Discussion

The treatment for mild and moderate osteoarthritis of the knee mainly referred to the conservative treatment included weight loss, physical exercise, nonsteroid anti-inflammatory agents, analgesics, and hyaluronic acid injection an injection of corticosteroid [1, 29]. The clinical use of PRP is becoming more frequent in the treatment of symptomatic KOA. To our knowledge, this is the first meta-analysis providing comprehensive insights into the efficacy and safety of PRP associated with several conservative treatments mentioned above, such as oral NSAIDs, intra-articular CS, intra-articular HA, and even intra-articular placebo.

Compared with saline solution as the placebo group, the IA PRP gained a lower VAS score at 6^th^ month, lower WOMAC score at 1^st^ month and 6^th^ month, and even higher IKDC subjective score after treatment. Because of the placebo effect and the increased efficacy of invasive treatment in patients, there were published scientific data on the effects of intra-articular saline solution injection on decreasing nociceptive pain in KOA patients [26]. Hence, IA PRP was significantly effective in pain relief and function improvement in the short term or mid-and-long term in this meta-analysis. Cartilage loss is a main course of KOA, and Elik et al. [26] reported that the PRP did not have any effect on cartilage thickness, but it adjusted joint homeostasis, cytokine levels, and decreased synovial hyperplasia, which were all considered as the pain reasons of the knee. To evaluate IA PRP's safety, there was no significant difference in adverse effect rate between the PRP and saline groups during the follow-up period. No severe side effect was found in the five studies comparing PRP versus saline [3, 9, 17, 20, 26].

Topical NSAIDs were shown to be effective for pain relief and function improvement in osteoarthritis as a first-line method [1]. Intra-articular injection therapies were all invasive treatments, and the application of oral NSAIDs served as a control group, could compare the efficacy between IA PRP and oral NSAIDs, and contrast the invasive treatment versus nonintra-articular treatment. It was showed the PRP group was associated with a lower WOMAC score at the 6^th^ month after treatment than the NSAID group (Figure S1) and proved that PRP is effective in the treatment of KOA patients, superior to oral NSAIDs, in the medium and long term.

When compared the VAS score at 2^nd^ and 6^th^-month follow-ups between the PRP group and the CS group, different studies [10, 16, 22] applied different VAS score models, including 10 mm VAS score and 100 mm VAS score, and we used a stand mean difference (SMD) to assess the results. There were no significant differences between the two groups in the short term (2 months, Figure S2), but the VAS score was significantly lower in the PRP group on the medium and long term (6 months, Figure S2) than the CS group. In contrast to the WOMAC score at the 6^th^-month follow-up, the PRP and CS groups did not show significant differences. Intra-articular corticosteroid injections are frequently used to treat acute or chronic inflammatory conditions, especially for patients who have not responded to oral or topical analgesics. The effects of CS are mainly anti-inflammatory, brought about by inhibiting inflammatory cytokines and blocking the pathways leading to their actions [16]. The IA PRP revealed a better duration in reducing pain than IA CS via comparing the VAS score at 6^th^-month follow-up, but not significantly superior to IA CS in pain reduction in the short term.

Hyaluronic acid, high molecular weight glucosamine, is provided viscoelasticity in the synovial fluid and extracellular matrix. The efficacy of HA treatment in improving osteoarthritis symptoms has been widely reported, and the clinical outcomes for patients with KOA are positive [30]. Also, several studies showed that the effect of intra-articular injection of HA or PRP depended on time [2]. In our meta-analysis, we found that there were no significant differences between the PRP group and the HA group at 1^st^ month after treatment in VAS score, WOMAC score, and IKDC subjective score, but the PRP group gained a lower VAS score at 12^th^ month during the follow-up and lower WOMAC score at 6^th^ month and 12^th^ month after the treatment. The PRP group's IKDC subjective score improved at the 6^th^ month and 12^th^ month during the follow-up. Di Martino et al. [25] considered that the biological intervention should have more effect on the intra-articular tissues and lead to better results at longer follow-up times [2]. To contrast the mechanism of PRP and HA in the change in KOA, the positive effects of HA may be attributable to improved lubrication on account of the viscoelasticity or improvement of the intra-articular environment via recovering the barrier between the synovial membrane and the articular surface; PRP provides growth factors that can be used to enhance tissue regeneration and promote concentrated anti-inflammatory signals. HA works as a lubricator, while PRP provides several factors to stimulate the synovial membrane and surrounding tissues [2]. In the aspect of safety, this meta-analysis revealed that there was no significant difference in adverse event rate between the PRP group and the HA group.

Injective therapies are very common and have repeatability over time, but repeated injections carry the risk of infective sequelae that could be devastating. Therefore, whether multiple injections of PRP are superior to single injection has clinical significance because fewer injections can reduce the risks associated with injections. The efficacy of multiple injections or a single PRP injection might differ at the pain reduction degree and action duration. We did not find significant differences in VAS score at 1^st^ month or WOMAC score at 3^rd^ month during the follow-up between the triple PRP group and the single PRP group in our meta-analysis. This result indicated the number of injections might not influence clinical effects in the short term. Simental-Mendía et al. [27] reported that the triple injection of PRP in patients with mild knee osteoarthritis was clinically more effective than the single application on long-term follow-up of 48 weeks. More high-quality, large-sample studies should be conducted to determine the long-term efficacy and safety of multiple injections of PRP versus single PRP injection.

Since PRP's preparation and formulation vary in different studies, and the PRP therapies are not identical and significantly different from each other. We further analyzed the included studies and summarized the preparation methods of 19 studies in which the authors had described the PRP purification protocol in detail (Table 2). PRP preparation's core technology is centrifugation [31], which is commonly used twice to separate erythrocytes and concentrate platelets, respectively [6, 13]. In studies included in our meta-analysis, 13 of them used the double-spin methodology to prepare PRP. Meanwhile, 6 of them used a single-spin methodology. The volume of collected blood before preparing PRP ranged from 8 mL to 150 mL. Nine studies reported using anticoagulants during the PRP preparation, and the most commonly used anticoagulant was citrated dextrose. Two studies stated that anticoagulants were not used in the preparation of PRP. Others did not report relevant information in their studies. Patel et al. [9] used a leucocyte percolator to filter white blood cells, and leucocyte count was zero in their PRP. Cole et al. [18] utilized a low-leukocyte PRP system, a single-spin system that concentrated platelets and separated red blood cells and white blood cells during the preparation process. Finally, we found that the most significant difference in PRP in each study was in leucocyte count. Among studies included in our analysis, 6 studies used leukocyte-poor PRP (LP-PRP), and 13 studies used leukocyte-rich PRP (LP-PRP). We conducted a subgroup analysis to ascertain whether there was a distinction between LP-PRP and LR-PRP in the efficacy of treating with KOA. As was shown in Figure 6, the LP-PRP group was associated with a higher IKDC subjective score at the 12th month after treatment than the HA group. Meanwhile, no significant difference was found between the LR-PRP group and the HA group on IKDC subjective score at the 12th month after treatment. The result revealed that LP-PRP might be more effective in improving functional outcome scores compared with LR-PRP. It has been noted that the presence of leucocytes in the space joint could generate a negative proinflammatory environment in OA cartilage [8]. Besides, more swelling and pain reactions have been reported when using LR-PRP [5, 8, 14]. Sundman et al. [32] reported that growth factor and catabolic cytokine concentrations were influenced by PRP's cellular composition. They found that platelets increased anabolic signalling, and, in contrast, leukocytes increased catabolic signalling molecules. However, other researchers mentioned limited evidence for comparing LP-PRP's clinical outcomes versus LR-PRP [33]. Further randomized trials are needed to assess further and to compare the efficacy of LP-PRP and LR-PRP.

There were some limitations to this meta-analysis. (1) Between-study heterogeneity remained high and unexplained across several indications. (2) Study sample sizes were small, further limiting the reliability of results inferred from the combined statistic. (3) Too many evaluation tools were used across the different studies such that the highest number of studies that used any single evaluation tool was six studies for the IKDC score at 6 months between the PRP group and the HA group. (4) Owing to the lack of sufficient extracted data, some of the outcomes could not be analyzed.

## 5. Conclusion

The current meta-analysis found that the PRP group had significantly effective pain relief and function improvement in the short term or mid-and-long term compared to the placebo group. As compared to the oral NSAID group, the intra-articular PRP group had a lower WOMAC score at 6 months. As compared to the intra-articular CS group, the intra-articular PRP group had a lower VAS score at 6 months. As compared to the intra-articular HA group, the intra-articular PRP group had a lower VAS score at 12 months and lower WOMAC score at 6 months and 12 months. There were no significant differences between the triple PRP group and the single PRP group in VAS score and WOMAC score in the short term. There were no significant differences in adverse event rates between PRP and placebo, as well as PRP and HA.

## Figures and Tables

**Table 1:** Characteristics of the included studies.

Study	No. trial/control (intervention)	Age (yr.) mean (trial/control)	VAS score at baseline (trial/control) mean ± SD	WOMAC score at baseline (trial/control) mean ± SD	IKDC at baseline (trial/control) mean ± SD
Cerza 2012 [13]	60/60 (PRP 4 IA/HA 4 IA)	66.5/66.2		76.9 ± 9.5/75.4 ± 10.7	
Filardo 2012 [14]	54/55 (PRP 3 IA/HA 3 IA)	55/58			50.2 ± 15.7/47.4 ± 15.7
Patel 2013 [9]	54/50/46 (PRP 1 IA/PRP 3 IA/saline 1 IA)	53.11/51.64/53.65	4.54 ± 0.613/4.64 ± 0.563/4.57 ± 0.620		
Raeissadat 2015 [15]	87/73 (PRP 2 IA/HA 3 IA)	56.85/61.13		39.5 ± 17.06/28.69 ± 16.69	
Filardo 2015 [6]	94/89 (PRP 3 IA/HA 3 IA)	53.32/57.55			52.4 ± 14.1/49.6 ± 13.0
Kavadar 2015 [4]	33/32/33 (PRP 3 IA/PRP 2 IA/PRP 1 IA)	55.2/54.9/53.6	8.4 ± 1.2/7.7 ± 1.2/7.7 ± 0.1	89.9 ± 1.7/81.6 ± 3.0/91.4 ± 2.0	
Cole 2016 [16]	49/50 (PRP 3 IA/HA 3 IA)	55.9/56.8			
Simental 2016 [27]	33/32 (PRP 3 IA/acetaminophen 500 mg tid po 6 weeks)	57.2/55.6	4.9 ± 2.4/5.9 ± 2.2	35.7 ± 19.5/35.5 ± 19	
Forogh 2016 [16]	24/24 (PRP 1 IA/corticosteroid 1 IA)	59.13/61.13	81.3 ± 13.4/77.8 ± 13.8 (100 mm VAS)		
Jubert 2017 [10]	35/30 (PRP 1 IA/corticosteroid 1IA)	65.56/68	75.14 ± 10.11/75 ± 9.38 (100 mm VAS)		
Görmeli 2017 [17]	39/44/39/40 (PRP 3 IA/PRP 1 IA+saline 2 IA/HA 3 IA/saline 3 IA)	53.7/53.8/53.5/53.5			40.4 ± 5/41.2 ± 6.1/40.6 ± 4.5/40.4 ± 4.3
Su 2018 [21]	27/25/30 (PRP 2IA+IO/PRP 2 IA/HA 5 IA)	50.67/53.13	7.09 ± 0.31/7.02 ± 0.27/7.04 ± 0.33	50.15 ± 1.10/50.17 ± 1.60/49.88 ± 1.54	
Ahmad 2018 [11]	45/44 (PRP 3 IA/HA 3 IA)	56.2/56.8	5.8 ± 1.9/6.1 ± 1.7		49.2 ± 14.9/47.2 ± 16.2
Uslu 2018 [22]	14/19/17 (PRP 3/PRP 1/corticosteroid 1)	60.4/62.3/62.8	4.1 ± 1.0/4.6 ± 0.7/5.4 ± 0.7	62.9 ± 4.2/58.1 ± 3.3/59.7 ± 3.2	
Lisi 2018 [19]	31/31 (PRP 3 IA/HA 3 IA)	53.5/57.1	6.28 ± 0.59/5.4 ± 0.36	36.96/3.33/28.48 ± 2.22	
Buendía 2018 [23]	33/32/33 (PRP 1 IA/HA 1 IA/Etoricoxib 60 mg qd po 52 weeks)	56.15/56.63/57.42	6.15 ± 1.1/6.06 ± 0.9/6.15 ± 1.2	42.57 ± 7.3/42.62 ± 7.3/42.66 ± 7.8	
Wu 2018 [20]	20/20 (PRP 1IA/saline 1 IA)	63.25/63.25		89.6 ± 8.1/72.0 ± 6.6	
Louis 2018 [24]	24/24 (PRP 1 IA/HA 1 IA)	53.2/48.5	4.8 ± 2.3/5.1 ± 2.2	36.5 ± 16.8/34.7 ± 21.8	
Lin 2019 [3]	31/29/27 (PRP 3 IA/HA 3 IA/saline 3 IA)	61.17/62.23		52.81 ± 18.14/52.67 ± 18.06/48.59 ± 16.92	35.71 ± 13.77/35.93 ± 12.71/33.3 ± 10.52
Di Martino 2019 [25]	85/82 (PRP 3 IA/HA 3 IA)	52.7/57.5			53.3 ± 14.3/50.3 ± 13.2
Elik 2019 [26]	30/27 (PRP 3 IA/saline 1 IA)	61.3/60.19	3.87 ± 2.14/4.93 ± 1.68	56.40 ± 18.71/57.04 ± 15.12	
Simental 2019 [27]	17/18 (PRP 3IA/PRP 1 IA)	60.1/54.6	4.3 ± 2.5/7.3 ± 2.1	41.4 ± 15.5/44.2 ± 19.7	
Huang 2019 [28]	40/40/40 (PRP 3 IA/HA 3 IA/corticosteroid 1 IA)	54.5/54.8/54.3	4.57 ± 0.61/4.54 ± 0.596/4.64 ± 0.543	48.19 ± 4.96/47.23 ± 5.37/46.58 ± 5.74	

**Table 2:** Summary of preparation and formulation of PRP in the included studies.

Study	Trial/control	Volume of collected blood	Anticoagulant	Centrifugal method	Leukocyte-poor PRP
Filardo 2012 [14]	PRP vs. HA	150 ml	Not reported	Double-spin methodology	No
Patel 2013 [9]	PRP vs. saline	100 mL	Citrate phosphate dextrose and adenine	Single-spin methodology	Yes
Raeissadat 2015 [14]	PRP vs. HA	35-40 mL	Not reported	Double-spin methodology	No
Filardo 2015 [6]	PRP vs. HA	150 mL	Not reported	Double-spin methodology	No
Cole 2016 [18]	PRP vs. HA	60 mL	Not used	Double-spin methodology	Yes
Simental 2016 [27]	PRP vs. acetaminophen	27 mL	Sodium citrate	Double-spin methodology	Yes
Forogh 2016 [16]	PRP vs. corticosteroid	20 mL	Citrate dextrose	Double-spin methodology	No
Jubert 2017 [10]	PRP vs. corticosteroid	60 mL	Citrated dextrose	Single-spin methodology	No
Su 2018 [21]	PRP vs. HA	45 mL	Sodium citrate	Double-spin methodology	No
Ahmad 2018 [11]	PRP vs. HA	8 mL	Not reported	Single-spin methodology	No
Uslu 2018 [22]	PRP vs. corticosteroid	18 mL	Citrate dextrose	Single-spin methodology	No
Lisi 2018 [19]	PRP vs. HA	20 mL	Citrate dextrose	Single-spin methodology	No
Buendía 2018 [23]	PRP vs. HA vs. NSAIDs	60 mL	Not reported	Double-spin methodology	Yes
Wu 2018 [19]	PRP vs. saline	10 mL	Not reported	Dingle-spin methodology	No
Louis 2018 [24]	PRP vs. HA	52.5 mL (for men) or 37.5 mL (for women)	Citrate dextrose	Double-spin methodology	No
Lin 2019 [3]	PRP vs. HA vs. saline	10 mL	Not used	Dingle-spin methodology	Yes
Di Martino 2019 [25]	PRP vs. HA	150 mL	Not reported	Double-spin methodology	No
Elik 2019 [26]	PRP vs. saline	10 mL	Sodium citrate	Double-spin methodology	No
Huang 2019 [28]	PRP vs. HA vs. corticosteroid	8 mL	Not reported	Single-spin methodology	Yes

## Data Availability

The datasets generated or analyzed during the current study are available from the corresponding author on reasonable request.

## Supplementary Materials

Supplementary 1 Supplementary Data Set 1: PRISMA 2009 Checklist.

Supplementary 2 Supplementary Data Set 2: Figure S1: forest plot analysis of WOMAC score at 6^th^ month between PRP and NSAIDs.

Supplementary 3 Supplementary Data Set 3: Figure S2: forest plot analysis of VAS score between PRP and CS.

Supplementary 4 Supplementary Data Set 4: Figure S3: forest plot analysis of WOMAC score at 6^th^ month between PRP and CS.

Supplementary 5 Supplementary Data Set 5: Figure S4: forest plot analysis of WOMAC score between PRP and HA.

Supplementary 6 Supplementary Data Set 6: Figure S5: forest plot analysis of IKDC subjective score between PRP and HA.
