# Supplementary figures and images for: Efficacy and Safety of Intra-Articular Platelet-Rich Plasma in Osteoarthritis Knee: A Systematic Review and Meta-Analysis
Source: Biomed Res Int. 2021 Apr 30;2021:2191926. doi: 10.1155/2021/2191926 (PMC8294028; doi:10.1155/2021/2191926)

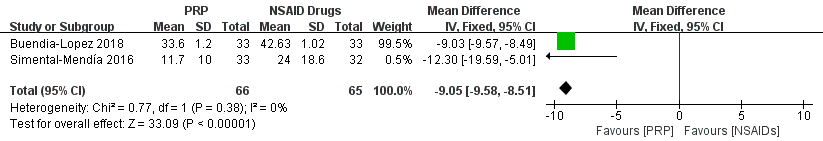


Figure S1 Forest plot analysis of WOMAC score at 6th month between PRP and NSAIDs.

Supplement: Supplementary 2 — Supplementary Data Set 2: Figure S1: forest plot analysis of WOMAC score at 6th month between PRP and NSAIDs. [file 2191926.f2.docx]

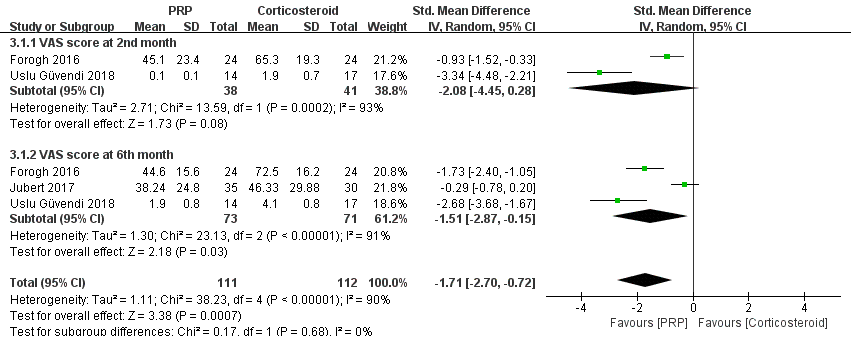


Figure S2 Forest plot analysis of VAS score between PRP and CS.

Supplement: Supplementary 3 — Supplementary Data Set 3: Figure S2: forest plot analysis of VAS score between PRP and CS. [file 2191926.f3.docx]

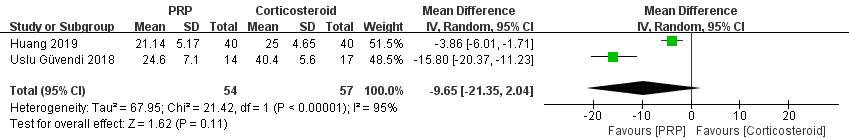


Figure S3 Forest plot analysis of WOMAC score at 6th month between PRP and CS.

Supplement: Supplementary 4 — Supplementary Data Set 4: Figure S3: forest plot analysis of WOMAC score at 6th month between PRP and CS. [file 2191926.f4.docx]

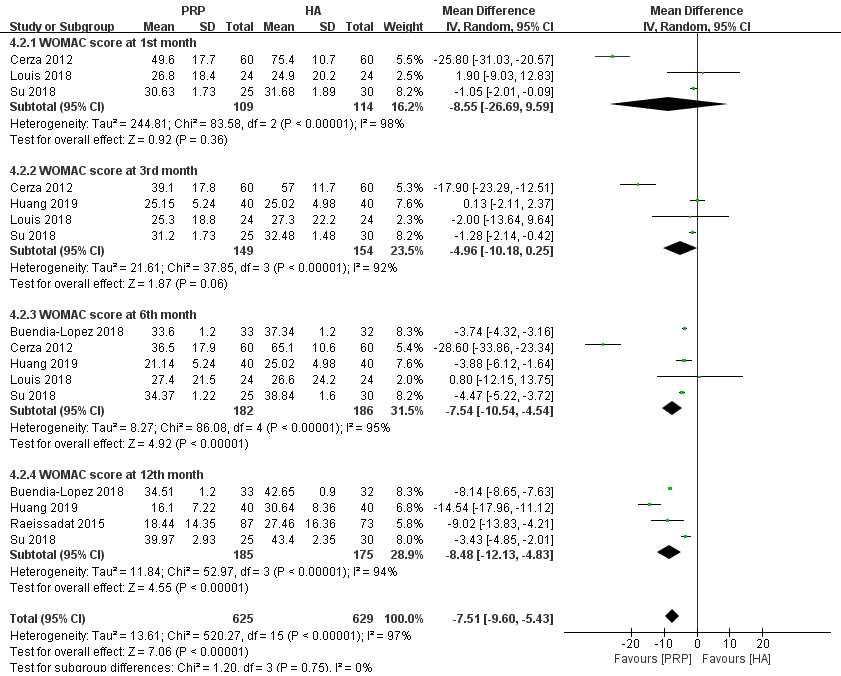


Figure S4 Forest plot analysis of WOMAC score between PRP and HA.

Supplement: Supplementary 5 — Supplementary Data Set 5: Figure S4: forest plot analysis of WOMAC score between PRP and HA. [file 2191926.f5.docx]

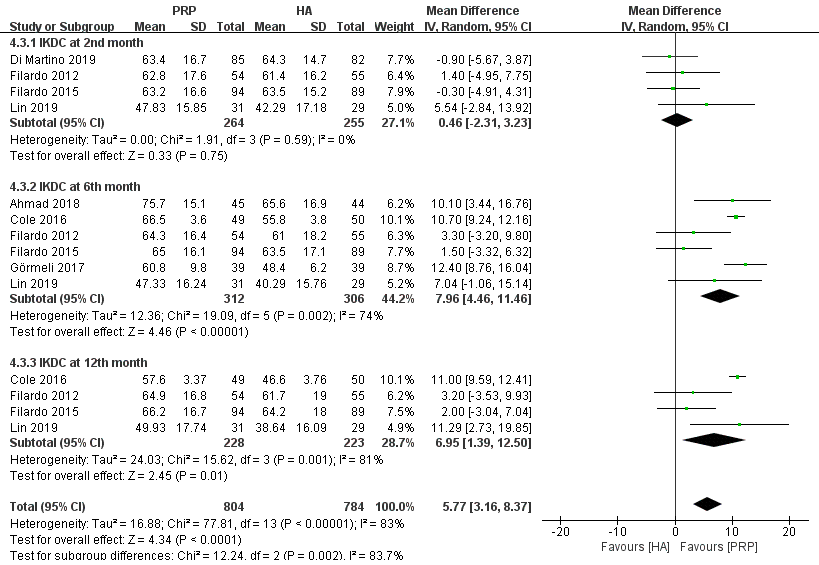


Figure S5 Forest plot analysis of IKDC subjective score between PRP and HA.

Supplement: Supplementary 6 — Supplementary Data Set 6: Figure S5: forest plot analysis of IKDC subjective score between PRP and HA. [file 2191926.f6.docx]
